# Induced tRNA Import into Human Mitochondria: Implication of a Host Aminoacyl-tRNA-Synthetase

**DOI:** 10.1371/journal.pone.0066228

**Published:** 2013-06-14

**Authors:** Ali Gowher, Alexandre Smirnov, Ivan Tarassov, Nina Entelis

**Affiliations:** Department of Molecular and Cellular Genetics, UMR 7156 Génétique Moléculaire, Génomique, Microbiologie (GMGM), CNRS - Université de Strasbourg, Strasbourg, France; Centro de Investigacion Principe Felipe and IBV-CSIC, Spain

## Abstract

In human cell, a subset of small non-coding RNAs is imported into mitochondria from the cytosol. Analysis of the tRNA import pathway allowing targeting of the yeast tRNA^Lys^_CUU_ into human mitochondria demonstrates a similarity between the RNA import mechanisms in yeast and human cells. We show that the cytosolic precursor of human mitochondrial lysyl-tRNA synthetase (preKARS2) interacts with the yeast tRNA^Lys^_CUU_ and small artificial RNAs which contain the structural elements determining the tRNA mitochondrial import, and facilitates their internalization by isolated human mitochondria. The tRNA import efficiency increased upon addition of the glycolytic enzyme enolase, previously found to be an actor of the yeast RNA import machinery. Finally, the role of preKARS2 in the RNA mitochondrial import has been directly demonstrated *in vivo*, in cultured human cells transfected with the yeast tRNA and artificial importable RNA molecules, in combination with preKARS2 overexpression or downregulation by RNA interference. These findings suggest that the requirement of protein factors for the RNA mitochondrial targeting might be a conserved feature of the RNA import pathway in different organisms.

## Introduction

Mitochondria are essential organelles of almost all eukaryotic cells and take part in several critical cellular processes. They contain their own genome and perform transcription and translation of their genetic material. However, the vast majority of biological macromolecules found in mitochondria are imported from the cytosol. For instance, the total number of mitochondrial protein species is about 850–900 whereas the mitochondrial genome codes for only 8 proteins in yeast and 13 ones in human cells, so all other proteins are imported from the cytosol. The mechanisms of protein import into mitochondria are described in detail and appear as universal for all eukaryotes [1], [2]. The situation is different for RNA: several types of small non-coding RNAs were suggested to be imported into mitochondria in different species, and the mechanisms of these processes are believed to be different in each case (see for review [3], [4], [5]).

**Figure 1:**
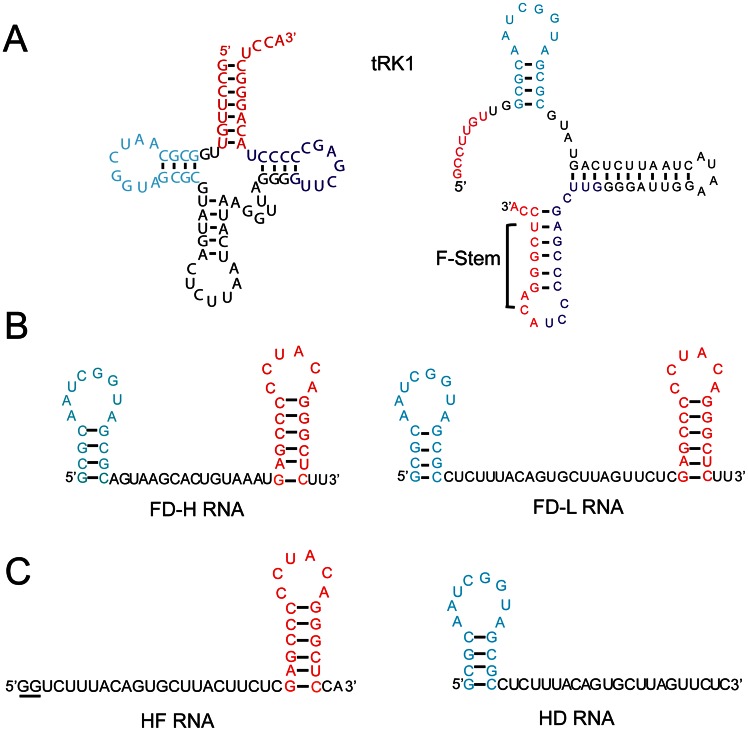
Predicted structures of the yeast tRNA^Lys^_CUU_ (tRK1) and small synthetic RNAs. (**A**) Two alternative structures of tRK1, as in [12]. The cloverleaf structure is shown at the left, the F-structure at the right. The tRK1 amino acceptor stem is in red, the D-arm in blue and the T-arm in purple. (**B**) Secondary structures of small synthetic “anti-replicative” RNAs composed of the tRK1 D-arm (in blue) and the F helix-loop structure (in red), separated by oligonucleotide stretches complementary to the heavy or light strands of human mitochondrial DNA [17]. (**C**) Truncated RNA molecules derived from FD-L RNA lacking either the D-arm of tRK1 (HF RNA) or the F-hairpin (HD RNA). The nucleotides added to the 5′-end of HF RNA to improve T7-transcription are underlined. For HF RNA, only one secondary structure (*dG* = −11.6 kcal/mol) was predicted by *Mfold*, for HD RNA, a structure with the minimal initial *dG* = −5.9 kcal/mol is shown.

In yeast *Saccharomyces cerevisiae*, the cytosolic tRNA^Lys^_CUU_ (further referred to as tRK1) is transcribed from a nuclear gene and then unequally redistributed between the cytosol (97–98%) and mitochondria (2–3%) [6]. The mitochondrial pathway was shown to be essential for mitochondrial translation at elevated temperatures, when the mtDNA-encoded isoacceptor tRNA^Lys^_UUU_ becomes undermodified at the wobble position of the anticodon and loses its capacity to recognize the lysine AAG codon [7]. The mitochondrial targeting of tRK1 in yeast *in vitro* and *in vivo* was shown to depend on the cytosolic precursor of mitochondrial lysyl-tRNA synthetase (preMSK1p), which serves as a carrier [8], [9], and the glycolytic enzyme enolase (Eno2p) [10], [11]. Analysis of conformational rearrangements in the RNA by *in-gel* FRET approach permitted to demonstrate that binding to the protein factors and the subsequent RNA import require formation of an alternative structure, different from the classic L-form tRNA model. In the complex with Eno2p, tRK1 adopts a particular conformation characterized by bringing together the 3′-end and the TΨC loop and forming a structure referred to as F-hairpin (Fig. **1A**) [12]. We suggested that only those RNAs that are able to form a stable alternative F-stem proceed to the mitochondrial import pathway involving specific interactions with the carrier protein, preMSK1p, and membrane receptors [13].

Exploiting these data, a set of small RNA molecules based on the F-hairpin sequence, with a significantly improved efficiency of import not only into yeast but also into human mitochondria *in vitro* and *in vivo,* have been constructed. This opened a possibility to design a new vector system capable to target therapeutic oligoribonucleotides into deficient human mitochondria [12]. So far, the RNA import is the only known natural mechanism of nucleic acid delivery into human mitochondria. Since many incurable neuromuscular diseases have been associated with mtDNA mutations, the RNA import represents a promising tool for the future gene therapy. The allotopic (nuclear) expression of recombinant tRNA molecules importable into mitochondria has been exploited to partially correct the pathogenic effect of mtDNA mutations in human cells [14], [15], [16]. Recently, we demonstrated that replication of mtDNA containing a pathogenic mutation can be specifically affected by RNA molecules bearing oligonucleotide stretches complementary to the mutated region. These molecules can be targeted into human mitochondria *in vivo* using artificially engineered RNA vectors based on the tRK1 alternative structure (**Fig. 1A, B**) [**17**]. To further develop and optimize this approach, we need to understand the molecular mechanism of RNA targeting into human mitochondria, especially the protein factors participating in this process. This question is addressed in the present study.

It was previously found that the synthetic transcripts of yeast tRNAs^Lys^ and a number of their mutant versions could be specifically internalized by isolated human mitochondria in the presence of yeast or human soluble cytosolic proteins, indicating that the human cell possesses the machinery needed for the tRNA mitochondrial import [18], [19]. We also suggested that the cytosolic precursor of human mitochondrial lysyl-tRNA synthetase (preKARS2) could replace its yeast homologue preMSK1p and serve as a carrier for tRK1 [19]. In human cells, a single *KARS1* gene codes for both mitochondrial and cytosolic lysyl-tRNA-synthetases which are translated from two mRNAs generated by alternative splicing [20]. Here we use abbreviations KARS2 and preKARS2 for the mature mitochondrial enzyme and its cytoplasmic precursor, correspondingly, and KARS1 for the cytosolic enzyme. Recently, another research group has demonstrated that the recombinant KARS2 can substitute preMSK1p in targeting tRK1 into isolated yeast and mammalian mitochondria in the presence of the yeast cytosol [21].

Here we show that preKARS2 has an affinity to tRK1 and artificial RNA molecules containing the structural elements which determine the tRK1 mitochondrial import. These molecules can be targeted into isolated human mitochondria in the presence of preKARS2 and mammalian enolase, thus demonstrating a similarity to the yeast system. Finally, the role of preKARS2 in the RNA mitochondrial import is, for the first time, demonstrated *in vivo*, in human cells transfected with tRK1 and artificial importable RNA molecules.

## Results

### PreKARS2 Binds tRK1 and Artificial Importable RNA Molecules

**Figure 2:**
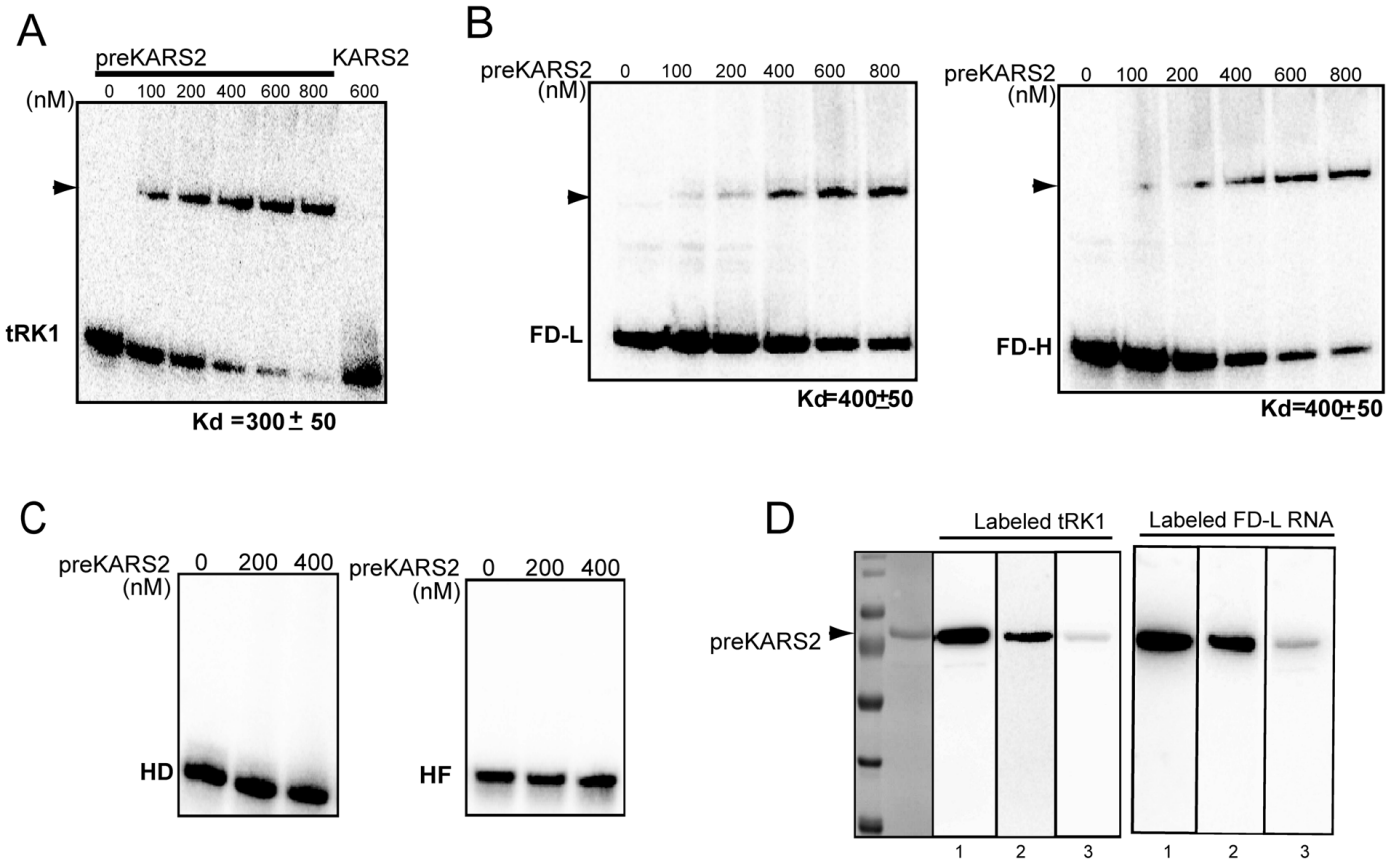
Interaction of the purified KARS2 and preKARS2 proteins with RNAs tested by EMSA. After incubation of ^32^P-labeled tRK1 (**A**), FD-L and FD-H RNAs (**B**) or the truncated HD and HF RNAs (**C**) with increasing concentrations of the recombinant proteins (indicated above the panels, in nM), the complex formation was visualized by autoradiography. In each assay, the bottom band corresponds to the free RNA species, the RNA-protein complex is marked with an arrowhead. The deduced dissociation constants (*K_d_*) for each RNA are given at the bottom of the panel (in nM). A representative of at least three independent experiments is shown for each RNA. (**D**) North-Western hybridization. Membrane stripes, containing equal amounts of preKARS2, were incubated with labeled RNAs (indicated above). Lane 1, no competitor added; lane 2, hybridization in the presence of 30× molar excess of nonspecific competitor (rRNA *E. coli*); lane 3, hybridization in the presence of 10× molar excess of nonlabeled RNA FD-R. Left panel represents the membrane stained with Ponceau Red.

To study the implication of the cytosolic precursor of human mitochondrial lysyl-tRNA synthetase (preKARS2) in the mitochondrial import of the yeast cytosolic tRNA^Lys^_CUU_ (tRK1), we first analysed the interaction of the recombinant preKARS2 with a T7-transcript of tRK1 by EMSA (**Fig. 2**), using labeled RNA and increasing concentrations of the protein, as described [10]. The apparent *K_d_* of the complex was estimated as 300+/−50 nM. Thus, the affinity of preKARS2 to tRK1 is only slightly lower than that of its yeast homolog, preMSK1p, with the apparent *K_d_* previously evaluated as 280+/−60 nM [9]. Noteworthily, the recombinant protein lacking the mitochondrial targeting pre-sequence predicted by Mitoprot [20] and thus corresponding to the mature mitochondrial enzyme KARS2 was not able to interact with tRK1 (**Fig. 2A**). This finding parallels our previous study suggesting a particular way of interaction between tRK1 and yeast preMSK1p which does not lead to the tRNA aminoacylation [8].

Previous analysis of RNA aptamers imported into human mitochondria permitted us to design short synthetic RNAs comprising two domains of the tRK1 alternative structure (**Fig. 1** **A, B**) and characterized by a high efficiency of mitochondrial targeting [12], [17]. The molecules referred to as FD-L and FD-H, containing the D-arm and F-hairpin parts of tRK1 separated by 17–22 nucleotides stretches, were able to form complexes with the recombinant preKARS2 with the apparent *K_d_* of 400+/−50 nM, indicating a lower but still important affinity to preKARS2 (**Fig.** **2B**). The specificity of the interaction was verified by North-Western hybridization in the presence of specific and nonspecific competitors (**Fig. 2D**). The data show that 30× molar excess of cold *E. coli* rRNA only partially decreased the interaction of preKARS2 with labeled tRK1 and FD-L RNA, whereas the 10× molar excess of cold FD-R RNA completely abolished this interaction.

To study more precisely the role of each of the two stem-loop RNA domains, we constructed truncated FD-L RNA molecules (**Fig. 1C**) lacking either the D-arm (HF RNA) or the F-hairpin (HD RNA) of tRK1. Neither molecule was able to interact with preKARS2 (**Fig. 2C**), indicating the importance of the simultaneous presence of the D-arm and the F-hairpin for the RNA affinity to preKARS2.

### PreKARS2 can Direct the RNA Import into Isolated Human Mitochondria

Previously, we suggested that preKARS2 might replace preMsk1p in the import of tRK1 into human mitochondria [19]. To demonstrate this directly, the *in vitro* import test was performed by incubating the proteins and the labelled RNA with purified mitochondria from HepG2 cells, as described [22]. We tested the recombinant preKARS2 in combination with rabbit enolase, since our previous study of the tRK1 import into yeast mitochondria had shown that yeast enolase recognizes the imported tRNA and favours its binding to preMSK1p [10].

**Figure 3:**
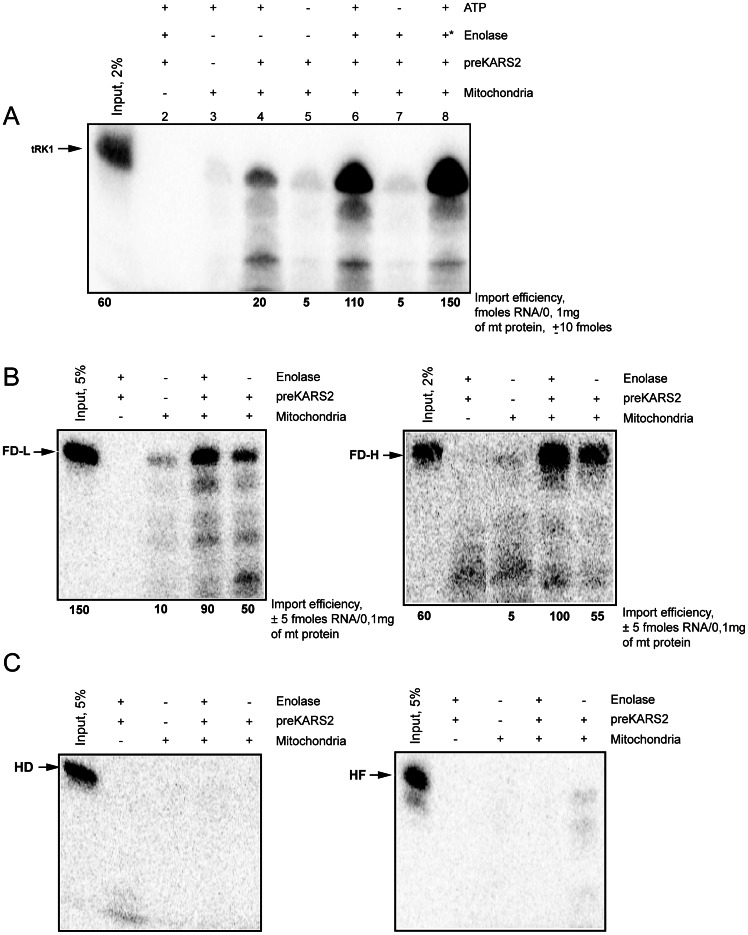
Import of RNA into isolated HepG2 mitochondria. Autoradiographies of RNA isolated from purified mitochondria and separated in denaturing 10% PAAG are presented. (**A**) Import of yeast tRK1, +*, yeast enolase was added instead of rabbit one. (**B**) Import of the small synthetic RNAs FD-L and FD-H. (**C**) Import of the truncated HD and HF RNAs. The name and position of the full-size RNA is indicated by an arrow on the left of each panel. Input, 2–5% of the RNA used for each assay (as indicated above the lane), corresponding to 60–150 fmoles of labeled RNA. Mitochondria (+) corresponds to the complete import assay, Mitochondria (-) to the mock import assay without mitochondria used as a control for non-specific protein-RNA aggregation. The RNA import efficiency was calculated by comparing the signal with the input and is indicated below each lane. A representative of three independent experiments is presented for each RNA, ±SD indicated.

Purified human mitochondria were not able to internalize the external tRK1 in the absence of protein factors (**Fig. 3**). Control reactions without mitochondria or in the absence of ATP (**Fig. 3A**) demonstrate that the proteins do not protect the RNA from nuclease digestion. Upon addition of mitochondria and the recombinant preKARS2, a portion of tRK1 and the small artificial RNAs FD-L and FD-H has been protected from nuclease degradation (**Fig. 3** **A, B**), thus indicating their import into the mitochondria. The amount of the imported RNA was determined by comparison of the band density of the protected full-size RNA isolated from the mitoplasts after the import assay with that of an aliquot of the input labelled RNA, as shown in Fig. 3. As it was demonstrated previously [18], [19], only a minor fraction (1–5%) of the tRK1 added to the import mixture is transported into the isolated human organelles, corresponding to the *in vivo* situation in yeast [6] [23].

The amount of the imported RNA increased upon addition of rabbit enolase to the import mixture in combination with preKARS2, however, the effect of enolase was dependent on the RNA structure. tRK1 was very poorly imported with preKARS2 alone but its import has been significantly improved upon addition of either rabbit or yeast enolase (**Fig. 3A**), demonstrating the interchangeability of the yeast and mammalian targeting systems. The recombinant human enolase (hEno1) had the same effect on the tRK1 import *in vitro* as the rabbit one (not shown).

In contrast to the situation with tRK1, the level of mitochondrial import of the FD-L and FD-H RNA molecules was rather high in the presence of preKARS2 alone and has only been slightly improved upon rabbit enolase addition (**Fig. 3B**). These data are in agreement with our model suggesting that only in the alternative F-conformation tRK1 acquires a high enough affinity to preMsk1p (**Fig. 1A**), and the RNA-chaperone activity of enolase is necessary for this structural rearrangement [12]. According to this suggestion, the presence of enolase should not be so important for the FD-L and FD-H RNA molecules, since they do not need the structural rearrangements for the interaction with preKARS2 and mitochondrial targeting.

As expected, the truncated RNA molecules HF and HD, which cannot interact with preKARS2, have not been directed into human mitochondria by this protein, independently of the presence of rabbit enolase (**Fig. 3C**).

### Implication of preKARS2 in the RNA Mitochondrial Import *in vivo*

**Figure 4:**
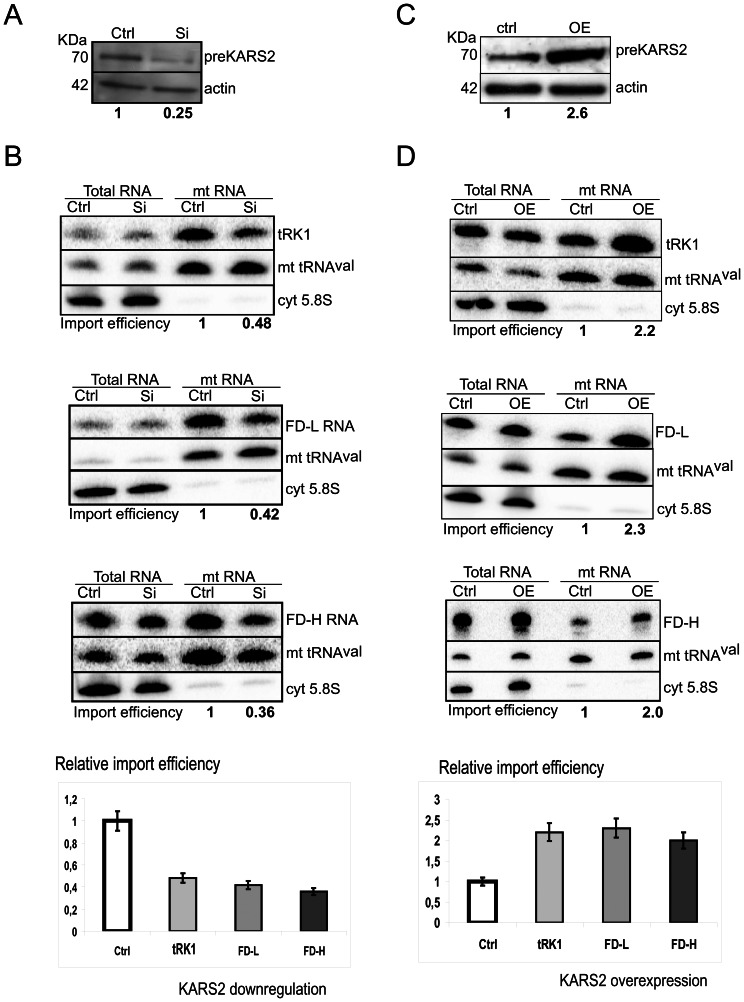
Implication of preKARS2 in the RNA mitochondrial import *in vivo*. (**A**) Western blot analysis of preKARS2 downregulation by RNA interference (Si). The level of preKARS2 in the cells transiently transfected with siRNAs against preKARS2 (Si) compared to the control cells transfected with a control siRNA (Ctrl) is indicated below the panel. The antibodies used for immunodecoration are shown on the right. (**B**) Northern blot hybridization of the total and purified mitochondrial (mtRNA) RNAs isolated from the control cells (Ctrl) and the cells transfected with siRNAs against preKARS2 (Si), in 32 h after transfection with tRK1, FD-L or FD-H RNA, as indicated. The hybridization probes are shown on the right. The mt tRNA^Val^ probe was used as loading control, and the cytosolic 5.8S rRNA probe was used to confirm the absence of cytosolic RNA contamination in the mitochondrial RNA preparations. The relative RNA import efficiencies, taken as 1 for the control cells, are shown below each panel (see Methods for the import efficiency calculation). For each RNA, the results of at least three independent experiments are shown at the lower panel, ±SD indicated. (**C**) Western blot analysis of preKARS2 overexpression (OE), the relative level of overexpression is indicated below the panel. Ctrl, control cells transfected with an empty vector. (**D**) Analysis of the *in vivo* import of tRK1 and the small synthetic FD-L and FD-H RNAs into mitochondria of the control cells (Ctrl) and the preKARS2-overexpressing (OE) cells in 48 h after transfection with the corresponding RNAs. All indications are as in **B**.

To compare the *in vitro* and *in vivo* import requirements, the role of preKARS2 in the mitochondrial RNA targeting was studied in cultured human cells. For this, we used the *in vivo* import assay on the cells transfected with RNA molecules, as described [12], [24]. To downregulate preKARS2, cultured human HepG2 cells were transiently transfected with a mixture of two siRNAs specifically designed against the part of the preKARS2 mRNA corresponding to the mitochondrial targeting sequence. Three days after the second transfection (see Methods section for details), a drop of more than 70% was observed for preKARS2 by Western blot (**Fig. 4A**). To evaluate the effect of the preKARS2 downregulation on the RNA import into mitochondria, the cells were transfected with purified T7-transcripts of tRK1, FD-L or FD-H. The whole cell RNA and mitochondrial RNA were isolated from the control and preKARS2-downregulated cells and analysed by Northern blot hybridization (**Fig. 4B**). The absence of signal in the mitochondrial RNA after hybridization with the probe against the cytoplasmic 5.8S rRNA indicates that the treatment of mitochondria with ribonuclease and digitonin removed all contamination by cytoplasmic RNA. The amount of tRK1 molecules internalized by the cells was quantified by Northern blot hybridization using known amounts of T7-transcripts loaded on the same gel as standards. By this approach, we could estimate that 10.8±0.5% of the tRK1 added to the cells were internalized and could be detected in the full-size form 48 h after transfection. This value corresponds to 2.6±0.2×10^6^ RNA molecules per cell, which number is in the range of most abundant cellular RNAs, for example, 5S rRNA, estimated previously as 3.6±0.5×10^6^ RNA molecules per cell [19]. The number of tRK1 molecules in the mitochondrial fraction corresponded to 4.6±0.4×10^4^ RNA molecules per cell, giving 2.5±0.3% of the molecules imported into mitochondria from the cellular pool, which perfectly correlates with our *in vitro* data.

We observed a clear difference in the mitochondrial RNA import between the control and preKARS2-downregulated cells: the tRK1 import decreased 2-fold, and a 2.5-3-fold reduction was observed for the small artificial FD-L and FD-H RNAs import (**Fig. 4B**).

To confirm the role of preKARS2 as a mitochondrial targeting factor for tRK1 and its derivatives, we tested the RNA mitochondrial import in cells overexpressing preKARS2. For this, we used HeLa Tet-Off cells transiently transfected with a plasmid expressing preKARS2 (generous gift of M. Mirande, Gif-sur-Yvette, France). In 48 h after transfection, a 2- to 3-fold increase of the preKARS2 protein amount in the cell extract was detected (**Fig.** **4C**), in agreement with previously published data [25]. The cells overexpressing the preKARS2 protein were transfected with tRK1, FD-L or FD-H, and the mitochondrial RNA import was analysed by Northern blot hybridization (**Fig. 4D**), compared to control cells transfected with an empty vector. The mitochondrial import of all three RNA molecules, tRK1, FD-L and FD-H, increased 2-fold in the cells over-expressing preKARS2, confirming that the amount of the RNA molecules penetrating into mitochondria in human cells depends on the level of the preKARS2 protein expression.

All the data presented above clearly indicate the role of the human mitochondrial lysyl-tRNA synthetase preKARS2 in the mitochondrial targeting of yeast tRK1 and the artificial RNA molecules containing two structural elements of the tRK1 alternative “import-active” fold, the D-arm and the F-hairpin.

### Mitochondrial Import of Truncated RNA Molecules is not Dependent on preKARS2

**Figure 5:**
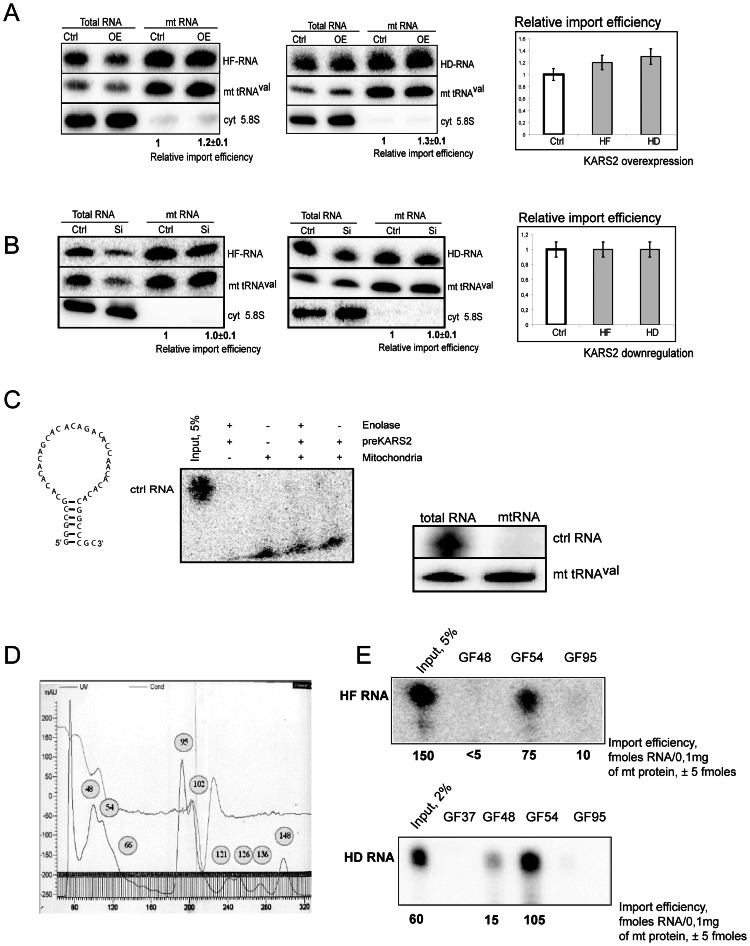
Import of small truncated RNAs into mitochondria. (**A**) *In vivo* import of the truncated HF (left panel) and HD (middle panel) RNAs in mitochondria of the control cells and the cells overexpressing preKARS2. (**B**) *In vivo* import of HF and HD RNAs in the control cells and the cells with downregulation of preKARS2. Hybridization probes are indicated on the right of the panels. Overexpression and downregulation of preKARS2 were confirmed by Western blot as in Fig. **4A, C**. The relative import efficiencies are shown on the right panels, ±SD calculated from three independent experiments. (**C**) Secondary structure (on the left) of the artificial control RNA, predicted by *Mfold*. The control RNA *in vitro* (middle panel, indications are as in Fig. **3**) and *in vivo* (right panel) import tests. (**D**) OD_280_ absorption profile of HepG2 proteins separated by gel filtration on a Sephacryl G-200 column. (**E**) Import of HF RNA (upper panel) and HD RNA (lower panel) into isolated HepG2 mitochondria in the presence of proteins from the gel filtration fractions indicated above the lanes. Input, 2–5% of RNA used for each assay. The RNA import efficiencies calculated by comparing with the inputs, in fmoles of imported RNA per 0.1 mg of mitochondrial protein, are given below each lane. On each panel, a representative of at least three independent experiments is shown, ±SD indicated.

Surprisingly, the small artificial RNA molecules containing either the D-arm or the F-hairpin (referred to as HD and HF, **Fig. 1C**), which were not imported into isolated human mitochondria *in* *vitro*, were internalized by mitochondria *in vivo* (**Fig. 5A, B**). A possible explanation of this discrepancy could be that our *in vitro* import conditions may not allow for a correct (predicted) folding of the short truncated RNA molecules. Nevertheless, the same RNAs internalized by cells, were able to be folded and imported into mitochondria.

To verify the specificity of our import test, we designed an artificial control RNA of a size similar to that of the HF and HD molecules (43 nt) but unrelated to yeast tRK1 and containing a short G-C stem and a long unstructured loop (**Fig. 5C** **).** This control RNA was not able to interact with the recombinant preKARS2 and to be imported into isolated human mitochondria in the presence of the purified proteins, preKARS2 and rabbit enolase (**Fig. 5C** **,** middle panel). Contrary to HD and HF RNAs, the control RNA was not detected in mitochondria of transfected HepG2 cells (**Fig. 5C,** right panel), indicating that not any short RNA molecule can be imported but only those containing the structural import determinants.

As it has been shown above, the HD and HF RNA molecules lack the capacity to interact with the recombinant preKARS2 and to be imported into isolated human mitochondria in the presence of preKARS2 and rabbit enolase (**Fig. 3C** **).** In agreement with these data, the *in vivo* import of these RNAs was not dependent on preKARS2, since no change in the amount of the RNA molecules transported into mitochondria was observed when preKARS2 had been transiently downregulated or overexpressed (**Fig. 5A, B**). This suggests implication of other protein factor(s) in the import of these RNAs into mitochondria *in vivo*.

To check if the mitochondrial targeting of the truncated RNAs is still dependent on protein factors, we isolated crude proteins from HepG2 cells, fractionated them by gel-filtration and tested the main peaks, each representing a mixture of many proteins, for their ability to direct RNA into isolated human mitochondria (**Fig. 5D**). We detected an efficient import of both truncated RNAs in the presence of one protein fraction (**Fig. 5E**), thus demonstrating that the *in vitro* mitochondrial import of the HD and HF RNA molecules is dependent on protein factors.

All presented data show that the RNA targeting into human mitochondria is a flexible process, allowing to import not only a full-size yeast tRNA but also its truncated versions. Import of tRK1 and the RNAs containing both tRK1 import determinants depends on the preKARS2 protein. Shorter truncated molecules were shown to be imported with a help of other, so far unidentified protein factor(s).

## Discussion

### PreKARS2 as a tRK1 Carrier to Human Mitochondria

In human cells, a subset of small non-coding RNA is imported into mitochondria from the cytosol [26], including some tRNAs (either in a natural or an artificial manner) [14], [27], the RNA components of RNase P and MRP endonuclease [28], [29], and 5S rRNA [30], [31]. Analysis of the cryptic tRNA import pathway, allowing the targeting of the yeast tRNA^Lys^_CUU_ into human mitochondria, performed in the present study demonstrated a similarity between the tRK1 import mechanisms in yeast and human cells. In yeast cells, preMSK1p and Eno2p were identified as the tRK1 mitochondrial targeting factors [10], [32]. A similar tRNA import pathway in human cells involves the orthologous proteins, preKARS2 and enolase. Moreover, the alternative folding of tRK1 as a determinant for the mitochondrial targeting in yeast [12] seems to be relevant in human cells as well, since we show that artificial RNA molecules containing two hairpin structures characteristic for the tRK1 alternative F-fold (**Fig. 1A**) can be efficiently imported into human mitochondria *in vitro* and *in vivo*, in a manner clearly dependent on the preKARS2 protein (**Fig. 3** **,** **4**).

Aminoacyl-tRNA-synthetases is a group of enzymes responsible for the specific attachment of amino acids to their cognate tRNAs, thus performing a key step of translation (reviewed in [33]). In human cells, one gene *KARS1* codes for both mitochondrial and cytosolic lysyl-tRNA-synthetases which are produced from two mRNAs generated by alternative splicing [20]. PreKARS2 possesses a specific N-terminal sequence of 49 amino acid residues, which is the only difference from KARS1 [20], [34]. The situation is opposite in yeast *S. cerevisiae* where the mitochondrial and cytosolic lysyl-tRNA-synthetases are encoded by distinct genes, *MSK1* and *KRS1* [35]. PreMSK1p plays an essential role in the mitochondrial targeting of the cytosolic tRNA^Lys^_CUU_ (tRK1) in yeast [8]. Previously, it has been demonstrated that human preKARS2 overexpressed in yeast can partially complement the growth defect associated with the loss of *MSK1* and can additionally facilitate the import of tRK1 into isolated yeast mitochondria [21]. Here we demonstrate the direct interaction of preKARS2 (but not of its mature form) with yeast tRK1 and the involvement of this protein in the tRK1 import into human mitochondria *in vivo*.

Recently and rather surprisingly, the mature mitochondrial enzyme KARS2 was shown to interact with the human cytosolic tRNA^Lys^_3_ with an apparent *K_d_* of 250+/−40 nM, but the presence of the mitochondrial targeting sequence in preKARS2 completely abolished the RNA-binding properties of the protein (*K_d_* >1 µM for preKARS2) [34]. Since in human cells no import of tRNA^Lys^ into mitochondria had been observed [26], the apparent discrepancy between these and our data clearly indicates a different mode of preKARS2 interaction with either the non-importable cytosolic tRNA^Lys^_3_ or the importable tRK1. This is in agreement with our hypothesis that only the alternative fold of tRNA can be recognized by the precursor of mitochondrial lysyl-tRNA-synthetase functioning as an RNA mitochondrial carrier. Thus, only yeast tRK1 and some specially designed RNA molecules capable to adopt the alternative conformation can interact with preKARS2 and be targeted into human mitochondria.

### RNA Targeting into Mitochondria: a Species-specific or a Universal Mechanism?

In general, each known case of RNA mitochondrial import appears somewhat special and thus not sufficient to establish a common RNA import mechanism [36]. The results of the present work, together with our previous data, enable us to revisit the paradigm of ‘extremely diversified’ RNA import pathways and to propose several rules which can be, if not universal, at least largely applicable to various RNA import systems.

Firstly, to be imported into mitochondria, an RNA should escape from the cytosolic channelling. According to this model, no free diffusion of macromolecules inside the cell is normally possible since all its components are well arranged in space and their movements are strictly regularized (channelled). Channelling was studied in detail on the example of tRNAs [37]. It was found that, starting from the very transcription event, a tRNA molecule is trapped in a standard sequence of events (processing, modification, nuclear export, translation) assured by protein components that function in a chain. They hand the tRNA from one to another avoiding its release into solution (reviewed in [38]). To make an RNA exit from the standard circuit, a well regulated deviation has to be provided by a special mitochondrial targeting factor which has a specific affinity to the cargo RNA. For example, in yeast cells, tRK1 is probably captured from the translation cycle by the glycolytic enzyme enolase and redirected to the mitochondrial surface [10]. The same event apparently exists in the artificial tRK1 import pathway in human cells, as we show here. In the case of the 5S rRNA import, this function is performed by the cytosolic precursor of mitochondrial ribosomal protein L18 (preMRP-L18) [31]. To assure the irreversible RNA withdrawing from the cytosolic channelling, the protein factor should possess a chaperone activity to change the RNA conformation, as it has been shown for tRK1 in the complex with yeast enolase [12] or for 5S rRNA and preMRP-L18 [31].

The next step of the pathway is a rapid discharge of the chaperone by another mitochondrial import factor. Examples of such a cascade were described in the yeast import mechanism where tRK1 is quickly transferred from enolase to the precursor of lysyl-tRNA synthetase [10]. A very similar case was observed for 5S rRNA in human cells where the mitochondrial enzyme rhodanese accepts 5S rRNA from preMRP-L18 [30]. For both mechanisms, a significant decrease in the apparent dissociation constant for the complex between the second protein factor and the RNA was found. Then, the second import factor works as a carrier transporting the RNA molecule into the mitochondria. The mechanism of RNA translocation across the double mitochondrial membranes is not yet understood. Most probably, it exploits the standard mitochondrial pre-protein localisation apparatus, since carriers usually have signals of mitochondrial localisation and it appears the most obvious way to reach the organelles. Nevertheless, one can not exclude alternative translocation mechanisms via different membrane channels [4], [29], [39].

Thus, for all RNA import systems in which the pre-mitochondrial (targeting) step of RNA import has been investigated, several universally present features can be outlined. Namely, in order to direct a cytosolic RNA to mitochondria one needs necessarily two protein factors, the first with a chaperone activity to withdraw the RNA from the cytosolic channeling, the second possessing the signal of mitochondrial localisation to target the RNA into the mitochondria. One of these proteins should be cognate, interacting with the imported RNA in a specific way and thus determining the selectivity of the RNA import (preLysRS for tRK1, preMRP-L18 for 5S rRNA). The other protein factor may be unrelated to RNA metabolism and hardly expected to participate in RNA transport, performing thereafter a “second job”, as enolase and rhodanese. Concerning enolase, many non-glycolytic “moonlighting” functions of this protein are known (reviewed in [40]). In *E. coli*, enolase is an integral component of the RNA degradosome; in yeast, it was identified as Hsp48 and participates in formation of vacuoles; enolase is found in the eye lens of many organisms and as a plasminogen-binding receptor expressed on the surface of a variety of eukaryotic cells. Thus, the tRNA import into mitochondria seems to be one of many different functions of this enzyme. The mitochondrial enzyme rhodanese is less studied, in fact, even its function is still not clear. We can hypothesize that this protein may also have multiple functions which can be switched by its cellular re-localization.

The common rules described here can be applied in search for RNA import pathways in various eukaryots. For instance, it appears that in plant cells, precursors of dually targeted cognate aminoacyl-tRNA synthetases combine both RNA targeting functions and thus may be the only essential tRNA import factors [36]. Probably, the same situation may be found in *Trypanosoma brucei,* where the cytosolic elongation factor eEF1a assures the specific targeting of almost all tRNAs to mitochondria [41].

### Mitochondrial Import of Small RNA Molecules

The general rules of RNA import formulated above presume certain flexibility of the pathway. Indeed, various RNA molecules able to interact with import factors can be targeted into mitochondria even in organisms naturally importing only a very restricted number of RNA species, as we see here for the RNAs FD-H and FD-L. On the other hand, various proteins might perform the function of RNA import factors in certain conditions. This possibility is clearly demonstrated in the present work since the short truncated RNA molecules HD and HF, which have lost the capacity to interact with preKARS2, apparently can be targeted into human mitochondria with the help of other, still unidentified protein(s). This hypothesis is also in agreement with a recent publication claiming that preMSK1p may be dispensable for the tRK1 import into yeast mitochondria [21]. One can suggest that in the yeast strain used in this study, lacking the *MSK1* gene and thus devoid of actively respiring mitochondria, the small amount of tRK1 detected in the pro-mitochondria could be imported by a backup pathway with a help of alternative targeting protein(s).

Recently, a subset of microRNAs, small non-coding RNAs that associate with Argonaute proteins to regulate gene expression at the post-transcriptional level, has been localized to human mitochondria [42], [43], [44], as well as the AGO2 protein [45]. At least a part of these miRNAs and their precursors were supposed to be imported from the cytoplasm by an unknown mechanism. It would be tempting to hypothesize that small structured RNA molecules, such as HD and HF, might be recognized by the machinery of the miRNA import and targeted to mitochondria by AGO2 and/or another components of the RNA-inducible silencing complex (RISC), which could thus perform the “second job” as mitochondrial targeting factors, similarly to the enzymes enolase or rhodanese. This exiting possibility remains to be explored in future studies.

## Materials and Methods

### Plasmids and Antibodies

Plasmid pDEST17 expressing human mitochondrial lysyl-tRNA synthetase (KARS2) was kindly provided by M. Sissler (IBMC, Strasbourg). To produce the precursor of mitochondrial KARS2 (preKARS2) protein, the Quick change mutagenesis kit (Stratagene) was used to insert the mitochondrial targeting sequence at the N-terminus of the mitochondrial KARS2 protein. A thrombin cleavage site and a 6× histidine tag at the N-terminus were deleted and a 6× histidine tag was inserted at the C-terminus using the same approach. For this, the following oligonucleotides were used:

### Mitochondrial Targeting Sequence Insertion

5′GCCACGCGGTTCTTTGACGCAAGCTGCTGTAAGGCTTGTTAGGGGGTCCCTGCGCAAAACCTCCTGGGCAG 3′

5′CTGCCCAGGAGGTTTTGCGCAGGGACCCCCTAACAAGCCTTACAGCAGCTTGCGTCAAAGAACCGCGTGGC 3′

### Thrombin Cleavage Site and 6× His Tag Deletion

5′ CTTTAAGAAGGAGATATACATATGTTGACGCAAGCTGCTGTAAGG3′

5′ CCTTACAGCAGCTTGCGTCAACATATGTATATCTCCTTCTTAAAG 3′

### His Tag Insertion at the C-terminus of preKARS2

5′ CAACAGTTGGCAGTTCTGTCCACCATCACCATCACCATTGAGACCCAGCTTTCTTGTAC 3′

5′ GTACAAGAAAGCTGGGTCTCAATGGTGATGGTGATGGTGGACAGAACTGCCAACTGTTG 3′

pTRE2hyg plasmid expressing preKARS2 and antibodies directed against the residues 25 to 42 of the preKARS2 protein described in [25] were kindly provided by Marc Mirande (Gif-sur-Yvette, France). Polyclonal antibodies against human actin were from Santa Cruz Biotechnology.

### Purification of the Recombinant preKARS2 Protein

To obtain the recombinant KARS2 and preKARS2 proteins, *Escherichia coli* strain BL21 codon plus (DE3)-RIL cells (Stratagene) were transformed with the pDEST17 plasmid. The transformed cells were grown in 500 ml of LB medium to a cell density corresponding to OD_600_ = 0.6, then the protein expression was induced for 2 h at 37°C by addition of 0.5 mM isopropyl β-D-1-thiogalactopyranoside (IPTG) to the bacterial culture. The cells were harvested by centrifugation at 6000 g for 10 min, lysed with 1 mg/ml of lysozyme on ice for 30 min and then sonicated thrice for 20 sec in the buffer consisting of 50 mM NaH_2_PO_4_, 300 mM NaCl and 20 mM imidazole. The cell lysate was centrifuged at 10,000 g for 15 min and the pellet was solubilized in the denaturing buffer consisting of 100 mM Tris-HCl (pH 8), 100 mM NaH_2_PO_4_, 10 mM imidazole and 8 M urea. This was followed by centrifugation at 12000 g for 15 min and the supernatant was applied to a Ni-NTA column (Qiagen) for 2 h at 4°C. After binding, the column was washed three times with the denaturing buffer containing 20 mM imidazole to eliminate weakly bound bacterial proteins. The recombinant preKARS2 protein was eluted from the column with 200 mM imidazole, refolded by stepwise elimination of urea and finally dialyzed against 50 mM Tris-HCl (pH 8), 300 mM NaCl and 40% glycerol and stored at –20°C. The purity of the protein was checked by sodium dodecyl sulfate-polyacrylamide gel electrophoresis (SDS-PAGE) with Coomassie blue staining.

The recombinant yeast enolase Eno2p was isolated as described previously [10]. Enolase from rabbit muscles was from Sigma-Aldrich.

#### Recombinant RNA modeling

To predict secondary structures of recombinant RNA molecules and estimate their free energies (*dG*), the *Mfold* program [46], [47] and IDT Sci-Tools OligoAnalyser 3.1 software [48] were used.

#### RNA synthesis and purification

The yeast tRK1 T7-transcript was obtained as described [32]. For small artificial RNAs, PCR amplification of the following oligonucleotides containing a T7 promoter at the 5′-end (underlined) was performed:

FD-L TAATACGACTCACTATA GCGCAATCGGTAGCGCCTCTTTACAGTGCTTAGTTCTCGAGCCCCCTACAGGGCTCTT

FD-H TAATACGACTCACTATA GCGCAATCGGTAGCGCAGTAAGCACTGTAAATGAGCCCCCTACAGGGCTCTT

HD: TAATACGACTCACTATA GCGCAATCGGTAGCGCCTCTTTACAGTGCTTAGTTCTC

HF: TAATACGACTCACTATA GGTCTTTACAGTGCTTACTTCTCGAGCCCCCTACAGGGCTCCA

RNA transcripts were obtained *in vitro* using the Ribomax kit (Promega). Following transcription, the DNA template was removed by digestion with RQ1 RNase-Free DNase (Promega). RNAs were purified by 12% PAGE with 8 M urea and eluted from the gel with the RNA extraction buffer containing 0.5 M CH_3_COONH_4,_ 10 mM Mg(CH_3_COO)_2,_ 0.1 mM EDTA and 0.1% SDS. The eluted RNA was precipitated with ethanol.

#### Electrophoretic mobility shift assay (EMSA)

Purified RNA was dephosphorylated with alkaline phosphatase (Boehringer Mannheim) and labeled at the 5′-end with γ-^32^P-ATP using T4 polynucleotide kinase (Promega). The labeled RNA was denatured at 100°C and then slowly cooled down to the room temperature. For RNA binding assays, the appropriate amount of protein and labeled RNA were mixed in 20 µl of the buffer containing 20 mM Tris–HCl pH 7.5, 150 mM NaCl, 10 mM MgCl_2_, 5 mM DTT, 10% glycerol, 0.1 mg/ml BSA and incubated at 30°C for 15 min. The mixture was separated by native 8% PAGE in 0.5×Tris-borate buffer (pH 8.3) and 5% glycerol [49], followed by Typhoon-Trio (GE Healthcare) scanning and quantification as described in [50].

#### North-Western blot hybridisation

Recombinant preKARS2 was loaded on 10% SDS-PAAG and blotted to nitocellulose membrane. The membrane was incubated in 0,1 M Tris-HCl, 20 mM KCl, 2,5 mM MgCl_2_, 0,1% Nonidet P40, pH7.5, at 4°C for 1 h with stirring, then washed several times with the same solution and blocked in 10 mM Tris-HCl, pH7.5, 5 mM Mg(CH_3_COO)_2_, 2 mM dithiothreitol, 2% BSA, 0,01% Triton X-100 for 5 min at 25°C. Then, the membrane was incubated for 2 h at 4°C in the import buffer without sorbitol, containing 1 nM [^32^P]-labelled RNA, as in [10], washed with the same buffer without RNA and analysed by Typhoon-Trio (GE Healthcare) scanning and quantification.

#### *In vitro* import assay

Mitochondria were isolated and verified for intactness as described [22]. The standard *in vitro* import assay into isolated mitochondria was performed as in [19]. For this, purified HepG2 mitochondria were incubated with radioactively labeled RNA and purified proteins in the import buffer: 0.6 M sorbitol, 20 mM HEPES-KOH (pH 7), 10 mM KCl, 2.5 mM MgCl_2_, 5 mM DDT and 2 mM ATP. For a standard *in vitro* assay, we add 3 pmoles of labelled RNA per 0.1 ml of the reaction mixture containing 0.1 mg of mitochondria (measured by the amount of mitochondrial protein). This corresponds to the 100% RNA input. After incubation for 15 min at 34°C, 50 µg/ml of RNase A (Sigma) was added and the reaction was incubated for additional 15 min to digest all unimported RNA. The mitochondria were washed three times with the buffer containing 0.6 M sorbitol, 10 mM HEPES-KOH (pH 6.7) and 4 mM EDTA, then resuspended in 100 µl of the same buffer and treated with an equal volume of 0.2% digitonin (Sigma) solution to disrupt the mitochondrial outer membrane, followed by purification of mitoplasts. The mitoplast pellet was resuspended in the solution containing 100 mM **CH_3_COONa, 10 mM MgCl_2_, 1% SDS and 0.05% diethylpyrocarbonate (DEPC), boiled for 1 min and RNA was extracted at 50°C with water-saturated phenol. RNA was precipitated with ethanol and** separated by 12% PAGE containing 8 M urea, followed by quantification with the Typhoon-Trio scanner using the Image Quant-Tools software (GE Healthcare). The amount of the imported RNA was determined by comparison of the band density of the protected full-sized RNA isolated from the mitoplasts after the import assay with an aliquot (2–5%) of the RNA input.

### Human Cell Culture, Overexpression and Downregulation of preKARS2

HeLa Tet-Off cells stably expressing the tetracycline-controlled transactivator (tTA) were purchased from Clontech Laboratories Inc. The HepG2 and HeLa Tet-Off cells were maintained in the Dulbecco modified Eagle’s medium (DMEM, Invitrogen) with high glucose (4.5 g/l) supplemented with 10% fetal calf serum, 100 µg/ml of streptomycin and 100 µg/ml of penicillin (Gibco). For induction of protein expression in HeLa Tet-Off cells, the Tet system approved fetal bovine serum from Clontech was used. The cells were cultivated in a humidified atmosphere at 37°C and 5% of CO_2._

For overexpression of preKARS2, HeLa Tet-Off cells were grown to the 60% confluency and transfected with the pTRE2hyg plasmid expressing preKARS2 [25] using Lipofectamin 2000 (Sigma) according to the manufacturer’s protocol. At the same time, the cells were transiently transfected with mitochondrially importable RNAs. After 48 h, the cells were analysed for the preKARS2 overexpression by Western blotting and for the RNA import by Northern hybridization.

To downregulate preKARS2, two 21-mer siRNAs corresponding to the mitochondrial targeting sequence of the human preKARS2 mRNA were synthesized. The sequences of the sense strands of these siRNAs are as follows: siRNA1∶5′ CAACTTGCTCCTTTCACAGCG 3′ and siRNA2∶5′ AAGGACAAGTCATTTTCTGAT 3′. As a negative control, a non-silencing siRNA (Ref: SR-CL000-005, Eurogentec) was used. Our optimized protocol consisted of two subsequent transfections: firstly, HepG2 cells were transfected in suspension with 40 nM of each siRNA using the RNAiMax transfection reagent (Invitrogen), according to the manufacturer’s protocol. 24 h later, the cells formed a monolayer and were transfected again with 40 nM of each siRNA using Lipofectamine 2000 (Invitrogen). The cells were grown for 40 h after the second siRNA transfection and then transfected with one of mitochondrially importable RNAs. In 3 days after the second siRNA transfection, the downregulation was analysed by Western blotting, and the RNA import by Northern hybridization.

### RNA Import Assay *in vivo*

For transfection of HepG2 and HeLa Tet-Off cells, 3 µg of RNA per 75 cm^2^ flask were used. Transfection was performed with the Lipofectamine 2000 reagent (Invitrogen), according to the manufacturer’s protocol. After 48 h, the cells were detached, mitochondria were isolated and purified as described above. The total and mitochondrial RNA were isolated with the TRIzol reagent (Invitrogen), separated by 12% PAGE containing 8 M urea and analysed by Northern blot hybridization with 5′-^32^P-labelled oligonucleotide probes:

anti-tRK1 (1–34): GAGTCATACGCGCTACCGATTGCGCCAACAAGGC to detect tRK1, FD-L, FD-H and HD RNA;

anti-HF RNA probe: TGGAGCCCTGTAGGG;

anti-mt tRNA^Val^ probe: GTTGAAATCTCCTAAGTG

and anti-cyt 5.8S rRNA probe: AAGTGACGCTCAGACAGGCA.

After quantification with the Typhoon-Trio scanner, the relative efficiency of the RNA import into mitochondria was calculated as a ratio between the signal obtained with the anti-tRK1 probe and that obtained with the probe against the host mitochondrial tRNA^Val^, as described previously [24]. Because it is rather difficult to normalize exactly the amount of mitoplasts isolated from various cell lines, we load on the gel the mitochondrial RNA isolated from the same number of cells, and then use the hybridization signals corresponding to the mitochondrial tRNA^Val^ as a loading control. Thus, we take into account not the absolute intensity of hybridization signals but the ratios between the signals corresponding to the imported into mitochondria tRK1 (or FD-RNAs) and the host mitochondrial valine tRNA’s gene transcript. To calculate the absolute import efficiencies for various RNAs, the total level of the RNAs in the transfected cells was taken into account. For this the relative import efficiencies were divided by the ratios calculated in the same way but for the total RNA preparations.

### Immunoblotting

For Western immunodecoration, cells were lysed in the Laemmli buffer (50 mM Tris-HCl, pH 6.8, 2% SDS, 0.1% β-mercaptoethanol, 0.01% bromophenol blue and 10% glycerol) for 10 min at 90°C, and 30 µg of protein was separated by 10% SDS-PAGE. The proteins were electroblotted onto a nitrocellulose membrane and probed with a primary polyclonal antibody against preKARS2 and a commercially available polyclonal antibody against actin (G2308, Santa Cruz Biotechnology). Bands were visualized with anti-rabbit or anti-goat secondary antibodies conjugated with horseradish peroxidase using the ECL Plus Western Blotting detection reagent (GE Healthcare).
